# Instructional guidelines and group discussion effects on new nurses’ competency regarding nursing care of preterm infants

**DOI:** 10.1016/j.heliyon.2024.e32586

**Published:** 2024-06-06

**Authors:** Abdulaziz Asiri, Faransa A. Ahmed, Abeer A. Almowafy, Rasha A. Mohamed, Wael G. Nouh, Aml S. Abdelrahem, Rehab H. Kafl, Manal F. Mohamed, Shimaa M. Moursy

**Affiliations:** a Department of Medical Laboratory Sciences, College of Applied Medical Sciences, University of Bisha, 255, Al Nakhil, Bisha 67714, Kingdom of Saudi Arabia; b Department of Nursing, College of Applied Medical Sciences in Alnamas, University of Bisha, 255, Al Nakhil, Bisha 67714, Kingdom of Saudi Arabia; c International Islamic Center for Population Studies and Research, Al-Azhar University, Cairo, Egypt; d Department of Nursing, College of Applied Medical Sciences, University of Bisha, 255, Al Nakhil, Bisha 67714, Kingdom of Saudi Arabia; e College of Applied Medical Sciences, King Faisal University, Saudi Arabia; f Pediatric Nursing Department, Faculty of Nursing, Minia University, Egypt; g Pediatric Nursing Department, Faculty of Nursing, Suez Canal University, Egypt; h Pediatric Nursing Department, Faculty of Nursing, Assiut University, Egypt

**Keywords:** Guideline, Group discussion, Competency, Nursing, Preterm infants

## Abstract

**Background:**

Premature birth was once one of the leading causes of infant mortality. Premature infants require improved medical and nursing skills from a highly competent nursing team.

**Purpose:**

This investigation aimed to assess the effects of instructional guidelines and group discussion on new nurses’ competency in preterm infants caring at the Neonatal Intensive Care Unit (NICU).

**Methods:**

A single-anonymized, two-group pre-test and post-test study design was accompanied in 2022. The study was accompanied for 50 newly graduated nurses concerned with competence in nursing management of preterm infants at the Neonatal Intensive Care Unit (NICU) in two hospitals: Al-Namas General Hospital, KSA, and the Pediatric Hospital of Assiut University, Egypt. Two groups were randomly selected from among the nurses to be trained on preterm infant nursing care competence either through an instructional guideline or group discussion. Nursing attitudes and practices were measured before and after training using questionnaires and checklists.

**Results:**

There was no significant difference between the instructional guideline group and the group discussion in the mean total score of attitudes (11.72 and 14.65, P = 0.455) and practices (14.36 and 14.80, P = 0.494) towards caring for preterm infants before intervention. While mean nurses’ attitude and practice scores increased significantly in both groups after the intervention, in the discussion group, nurses' practice scores increased significantly (p = 0.001). Still, there were no significant changes in the instructional guideline group (P = 0.202).

**Conclusion:**

Both methods were effective on the newly graduated nurses' attitude; however, the instructional guidelines did not affect their practice regard nursing care of preterm infants. However, group discussion training techniques can effectively improve the nurses’ attitude and practice toward nursing care of preterm infants.

**Relevance to clinical practice:**

The group discussion training method should be the approved and proven method for hospitals to train new nurses to increase clinical practices, especially during nursing care for premature infants, because this method has proven its worth in increasing the skill of nurses. It enables a group to talk about a specific subject and exchange personal stories. This approach involves a group leader facilitating communication and interaction between participants.

**Table:** 

What is known about this topic. Instructional guidelines and group discussion are effective methods for nurses' training to increase nursing skill competency during nursing work, so the authors need to compare instructional guidelines and group discussion to determine what's the most effective training method for newly graduated nurses that can maintain the competence of nurses' work at the neonatal intensive care unit. Standardized guidelines aid in enhancing the competence of nurses in clinical settings. Furthermore, because group discussions are collaborative and less expensive, they are employed increasingly often in healthcare settings. It enables a group to talk about a specific subject and exchange personal stories. What this paper adds. Group discussion techniques are the most satisfactory training methods for newly graduated nurses at the NICU. They can enhance newly graduated nurses' attitudes towards caring for preterm infants, which increases their nursing skill competency compared to training by instructional guideline technique. A key component of an effective group discussion is a safe space where people feel comfortable sharing their thoughts and experiences.

## 1 Introduction

Premature birth is a global epidemic, with an estimated 15 million births annually worldwide [1]. It is the leading cause of death worldwide and a significant cause of long-term loss of life. Of the 2.5 million neonatal deaths worldwide, 75 % are due to premature birth and its problems. The greatest of these deaths could potentially be prevented by improving the availability of qualified healthcare providers, the value of antenatal and postnatal care, and the care of small and sick newborns. The World Health Organization (WHO) defines premature birth as birth before the end of the 37th week of gestation [2].

Because these infants were born too early, they might have problems now and in the future that need to be taken care of. In places where there aren't enough resources, like doctors and nurses who know how to help these infants, they might not get the care they need and might not survive [3]. Preterm birth rates are increasing worldwide, but most notably in sub-Saharan Africa, including Tanzania. A global review of evidence suggests that an increase in preterm infants means a growing need for investment in quality preterm care [4].

Nurses are the primary caregivers of preterm infants, but most available studies have examined nurses' knowledge. No issues or solutions were identified. Understanding the current state of knowledge about the care of preterm infants and the factors that influence it will bring us one step closer to improving care that takes into account the morbidity and mortality burden of preterm birth [5].

A study by Lawn et al. reported that each newborn's basic set of care packages includes support for immediate and exclusive breastfeeding, thermal care, and umbilical cord and skin hygiene care. Rapid neonatal resuscitation is very important for infants who are not breathing at birth. Innovative solutions are urgently needed around the world to prevent premature births. For example, improved pregnancy and obstetric and neonatal care are needed to increase survival [6].

The essential nursing techniques employed in the neonatal intensive care unit to offer mothers of preterm newborns both practical and emotional support. These comprised telenursing, infant personalized developmental care and assessment programs, skin-to-skin care, parent support and education programs, interpersonal psychotherapy, and spiritual care [7].

Family participation in care decision-making, collaboration (inter-professional collaboration with family, family involvement in regulating and implementing care plans), respect and dignity for the family (importance of families' differences, recognition of families' tendencies), and knowledge transformation (information sharing between healthcare workers and family, complete information sharing according to family learning style) are all recognized characteristics of family-centered care in the neonatal intensive care unit. In addition, family-centered care benefits the newborns, families, nursing staff, and the organization. Family-centered care was a complete and all-encompassing strategy to providing care for neonates in the critical care unit. Enhancing the relationship between parents and their newborn, preventing infections, releasing the child earlier than usual, having an impact on the development of the baby's brain, raising staff and parent satisfaction, demonstrating the competence of parents in providing care for their newborn, creating a sense of home, cutting down on new medical costs, and enhancing safety [8].

Nurses who specialize in developmental care employ instructional principles and group discussions to optimize neurological development and minimize long-term behavioral and cognitive issues in newborns. Additionally, premature newborns now have lower fatality rates thanks to advancements in perinatal care, although their morbidity rates are still very high [9].

Nursing support on the unit is very important in the reduction of mental health problems that parents may experience, and nurse support in enabling parents to undertake aspects of care with their infants is crucial [10].

Nurses on neonatal units have greater success when supporting parents if they are clear about the rationale of the task they are helping parents learn [11]. Nurses are important in supporting breastfeeding development [12].

Nursing intervention for preterm infants included providing respiratory support, assessing heart sounds for murmurs, assessing pulse and perfusion, monitoring blood pressure, heart rate, and pulse pressure, and providing adequate hydration. Maintaining a neutral thermal environment, preventing infections, assessing readiness for selected procedures, providing appropriate stimulation according to the infant's condition and readiness, and promoting supine flexion using blanket rolls, the neonatal blanket, through the use of swaddles and cradles, rolls against the body and feet of the newborn to promote parent-neonatal bonding and to initiate phototherapy if necessary [13].

One way that it can influence nurses' awareness is through education. Preparatory training helps new nurses adapt and ensure quality service and patient safety. Training new pediatric nurses in China mainly focuses on developing critical care professionals and stratified training, which can often increase psychological pressure and hinder professional development [14].

Group discussion technique is used frequently in healthcare settings because it is less costly and collaborative. It enables a group to talk about a specific subject and exchange personal stories. This approach involves a group leader facilitating communication and interaction between participants. According to a study by Mirlashari et al. (2020), moms of premature children in the NICU can effectively reduce stress and enhance their coping mechanisms by participating in affordable, creative group talks about their babies with the healthcare team [15].

With proper preparation, instructional guidelines can also be used to learn subjects from different groups, such as patients, nurses, and nursing students. Using instructional guidelines significantly improved nurses' knowledge and practices regarding the care of high-risk neonates, according to a study by Mohammed and Abou Zed (2019) assessing the impact of instructional guidelines on nurses' performance regarding the care of high-risk neonates [16].

Nevertheless, no research has compared the effects of group discussions and guidance guidelines for novice caregivers on the nursing abilities of preterm infants. In light of these disparities, as well as the dearth of research-based lessons regarding the impact of training techniques on assessing nursing competence in preterm infants in a clinical setting, The researchers observed that some nursing students graduate without clinical exposure to the NICU because some hospitals only provide restricted pediatric clinical placements for nursing students to prevent infections in newborns. Students find it difficult to gain professional competencies through real-world experience as a result. After graduation, new nurses lack clinical experience in a range of unusual conditions that may develop in the NICU and are ill-prepared to manage emergency clinical situations. It has been noted that nurses, in particular, who are inexperienced in identifying and reacting promptly to emergencies, tend to take longer when dealing with high-risk infants [17].

Developing standard principles on the activities of NICU nurses and using them as a basis for practice in nursing education is necessary to ensure that new nurses are retained, trained competently in a situation where nursing education for premature infants is limited, and supported in their professional growth. Therefore, this study intends to apply a program through guidance guidelines and group discussions to enhance NICU nurses’ competence in providing care for preterm infants and to verify the effects of instructional guidelines and group discussions on the competency of new nurses.

## 2 Aim of the study

This study aimed to compare the effects of instructional guidelines and group discussion on new nurses’ competency in caring for preterm infants.

### 2.1 Research hypotheses

The following hypotheses were tested.

• The implementation of group discussion will significantly improve newly nurses' competency in caring for preterm infants more than training by an instructional guidelines technique in participating hospitals.
• The attitude of newly graduated nurses toward nursing care of preterm infants will significantly increase through group discussion rather than instructional guideline technique.
• The practice of new graduated nurses toward nursing care of preterm infants will score higher in terms of clinical teaching competence by group discussion than instructional guideline technique.

### 2.2 Research questions

• How do instructional guidelines versus group discussion affect new nurses' competency regarding nursing care of preterm infants?
• Which of the two methods—group discussion and instructional guidelines—is more successful?

### 2.3 Study design

This study used a single-anonymized, two-group, and design of pre-test and post-test. The study was accompanied from December 1st, 2022, to May 1st, 2023.

### 2.4 Materials and subjects

Participants of the study were newly graduated nurses at the Neonatal Intensive Care Unit (NICU) at Al-Namas General Hospital, KSA, and the Pediatric Hospital of Assiut University, Egypt.

A prior investigation contrasted the impacts of multimedia and brochures on pediatric nurses' knowledge, with a mean and standard deviation of 44.74 ± 3.4 and 40.74 ± 6.4 for the experimental and control groups, respectively [18]. All 50 newly graduated nurses were in their training year, so we did not sample for this study. A purposive sample of 50 newly graduated nurses was included in the study. They were assigned to two groups: an instructional guideline group and a group discussion group. A simple random sampling technique split the nurse list into two groups 25. The education department at Al Namas General Hospital, KSA, and Assiut University Children's Hospital, Egypt, assisted us in flipping a coin to determine which group would receive which teaching strategy (a set of guidelines or group discussions).

Inclusion criteria were newly graduated nurses who had completed at least 2 weeks of clinical training, had no prior training in caring for preterm infants, and were accepted to participate in the study.

The exclusion criteria for a decision to discontinue the study included nurses who had worked as pediatric nurses for more than two years or had transferred from another hospital; the absence of graduated nurses in the pre- or post-study stages of clinical training; or refusal to participate in this study.

### 2.5 The procedure

• Each subgroup nurse was required to complete four months of clinical training as per the schedule made by the training office.
• All supervisors agreed to work with the research team but were unaware of the research goals.
• They were assembled for an information session the day before formal clinical training for nurses began.
• Then, the nurse trainer organized for this session educated the nurses that their connections with premature infant care would be under study sometimes, without telling the particular time.
• The researchers managed confounding factors that can affect the teaching process, such as selected satisfactory free time and an adequate physical environment from lighting, ventilation, and satisfactory temperature.
• The two groups were to collect the data in parallel with the distribution of the researcher's instructional guidelines and group discussion interventions.
• The usual training was applied to newly hired nurses in the NICU previously at selected hospitals, as theoretical lectures were explained in the classroom by neonatal nursing specialists through PowerPoint data shows for all nurses at the same time without feedback from the participants. So, the researchers applied instructional guidelines and group discussion as effective methods to increase nurse's interest during training to improve their feedback, which can increase their performance competence.

### 2.6 The pre-test

• During clinical training, two investigators were trained to detect and document communications between nurses and preterm infants.
• A member of the research staff observed each nurse in each group. All pre-test observations were done secretly during the initial week of clinical instruction in the NICU.
• Once each subgroup achieved a practice pre-test, in agreement with the clinical instructor(s), The subgroup was assembled by the first researcher for a quick meeting in order to conduct the attitude pre-test.

### 2.7 Construction of an educational intervention session

• On Day 1 of the 2 nd week of clinical training, a 2-h group discussion was held on preterm care, its concepts and principles, and the consequences of respecting or ignoring competence. A subgroup of the first intervention group became an assigned group discussion method.
• All group discussion sessions were moderated by the first authors, who had prior training to facilitate group discussions.
• Strategies were used by experienced clinicians to manage the group.
• After welcoming and explaining the purpose of the session and the instructions for group discussion, each nurse received a paper with the session's topic and four instances of clinical practice. Each situation was associated with one of the competence domains in the care of preterm infants (i.e., tube feeding, warmth, hygienic care, phototherapy care, oxygen therapy, etc.).
• After every situation, there is a 4-select question requested newly graduated nurses: ‵‵Which area of competence did the case present relate to in caring for preterm infants?'' The teacher was given 30 min of study time. Review, evaluate, and answer relevant questions about four clinical cases. The session facilitator then gave a short speech on the concept of preterm infant care.
• A group discussion was then started in which nurses presented and discussed their opinions, observations, and experiences in maintaining competence in caring for preterm infants in the NICU. The facilitator invited the nurses to provide a summary of the meeting at the end.
• On the first day of the second week of the NICU clinical apprenticeship, each new nurse in the five subgroups of the instructional guidelines intervention received recommendations regarding their competency in nursing preterm infants.
• Newly graduated nurses were required to review the guidelines and return them to the investigator after one week. This instructional guide includes the concept of competence in preterm care, its implications, areas, outcomes, and the four competence domains in preterm care: tube feeding, warmth, hygienic care, phototherapy care, and oxygen therapy. These cases were the same as those presented in the group discussion.
• As in the first group, at the conclusion of each case, four-choice questions were posed to recently graduated nurses: ‘Which domain of preterm infant care was the offered situation related to?'
• The instructional guideline was developed through a literature review. Its information was validated by two faculty members from the Applied Medical Sciences in Al-Namas at the University of Bisha, three members from the pediatric nursing department at Assiut University, Egypt, and two pediatric nursing specialists from Al Namas General Hospital, KSA, in addition to three pediatric nursing specialists from Assiut University Children's Hospital, Egypt.

### 2.8 The post-test

• The first group started their post-test 14 days after their discussion, and the second group started 14 days after they returned their instructional guidelines.
• The post-test motor observations of each nurse were performed confidentially by the observers who performed the pre-test observations. After completing the post-test practice in each subgroup, the first investigator brought the subgroups together for a short session to finish the attitude post-test.

## 3 Instruments

### 3.1 Three parts of instruments were used

1. 1. The first section asked questions about age, gender, marital status, level of interest in the field of preterm care, and conflicts observed as a result of incompetence in preterm care in the NICU clinical setting.
2. The second part was a 34-item questionnaire to assess the newly graduated nurses' attitudes about nursing care competence for preterm infants. This part was adapted from **Smith et al. (2017)** [19] and was prepared by the researcher. This questionnaire addressed the main areas of focus for preterm infant nursing care, including tube feeding (6 items), warmth (7 items), hygienic care (8 items), phototherapy (6 items), and oxygen therapy (8 items). It has been constructed. An aspect related to developmental support care and pain management approaches for preterm infants is excluded because programs for preterm infant development care are not yet widely available in the study's setting.

All items are answered on a 3-point Likert scale, containing disagree (=-1), take no idea (=0), and agree (=1). After that, the total score fell between −34 and +34. A higher score means a healthier attitude. Ten pediatric nursing specialists confirmed the contented validity; the CVI was between 0.85 and 0.94. The contented validity ratio was >0.62. The reliability of the questionnaire was determined by a test-retest of 5 nurses (excluded from the study population) with a 2-week follow-up and a Spearman correlation coefficient of 0.70.

3. The third section of the tool included a checklist for evaluating ‘nursing practice’ in the care of premature infants. Established through a literature review [19,20] and validated by investigators, this checklist comprises of 11 items on the main aspects of preterm infant care, including tube feeding (5 items), warmth (4 items), hygienic care (6 items), phototherapy (4 items), and oxygen therapy (4 items). All items are scored on a 2-point scale. Compliance (=2) and non-compliance (=1) are included in the total score. The total score ranges from 11 to 22. A higher score indicates better practice. Ten pediatric nursing specialists verified the content and validity of the checklist. The CVI ranges from 0.95 to 0.98, and the CVR ranges from 0.62 to 0.62. The reliability of the checklist was evaluated using the inter-rater method.
4. To do this, the first investigator and the pediatric nurse educator simultaneously administered the checklist to 10 newly graduated pediatric nurses. 0.864 was the agreement coefficient.

### 3.2 Analytical statistics

Version 11.5 of SPSS SPSS Inc., Chicago, IL, USA Standard Deviation, Mean, Percentage, and Frequency are the Descriptive Statistics in SPSS. Test of Kolmogorov-Smirnov The data's normal distribution T-test in pairs Mean attitude scores from the Wilcoxon signed rank test before and after intervention separate samples attitude vs. practice *t*-test mean scores for both groups *P*-value less than 0.05.

## Ethical approval

The College of Applied Medical Sciences in Al-Namas Ethics Committee accepted the study at the University of Bisha, KSA. The study was also approved by the Ethics Committee of Al-Namas General Hospital and the Pediatric Hospital of Assiut University, Egypt. Before the study started, all recently graduated nurses completed a written informed consent form and were made aware of its aim. They were also told that even though they would be surveilled, the results would be kept private and would not affect how well they performed in their clinical training.

This study adheres to the highest ethical standard, as the ethical committee approved the study protocol of the IRB at the University of Bisha with the code number (UBCOM-RELOC H-06-BH-087)/0608.23).

## 4 Results

Among the 50 newly graduated nurses, 82 % were female, 94 % were single, and their mean age was 20.45 ± 1.35 years. 60 % of the studied nurses worked at the Neonatal Intensive Care Unit of the Pediatric Hospital of Assiut University in Egypt, and 40 % of them worked at Al-Namas General Hospital in KSA. The two groups were similar regarding demographic characteristics (Table 1). The current study demonstrated that nurses participating in group discussions during training were more satisfied and interested than nurses using instructional guidelines.

**Table 1:** The sociodemographic characteristics of newly graduated nurses in group discussions and instructional guideline techniques (No = 50).

Items	Groups			
	Instructional Guideline		Group Discussion	
	No.	%	No.	%
**Gender**				
Female	**21**	**84**	**20**	**80**
Male	**4**	**16**	**5**	**20**
**Marital Status**				
Single	**23**	**92**	**24**	**96**
Married	**2**	**8**	**1**	**4**

Table 2 shows a relation between of the mean and standard deviation of newly graduated nurses' attitudes regarding nursing care of preterm infants in an instructional guidelines group and group discussion techniques. Significant differences (P = 0.022) were observed between the two groups (instructional guidelines group and group discussion) in nurses' attitudes and newly graduated nurses' practices. There were higher attitudes (31.48 ± 4.83) among newly graduated nurses regarding the nursing care of premature infants in the instructional guidelines group compared to the group discussion (23.36 ± 8.99) techniques post-intervention. Significant differences (P = 0.357) were not found between the instructional guidelines group and group discussion techniques regarding practice, but an increase in the mean and standard deviation in practice (18.17 ± 2.54) of newly graduated nurses regarding the nursing care of premature infants in the group discussion techniques compared to the instructional guidelines techniques (15.49 ± 2.44) was found post-intervention.

**Table 2:** Relationship between mean and standard deviation of newly graduated nurses' attitudes regarding nursing care of preterm infants in an instructional guidelines group and group discussion techniques.

Assessment time	Groups	
	Instructional Guideline	Group Discussion
**Attitude**		
Pre-intervention	11.72 ± 7.09	14.65 ± 10.33
Post-intervention	31.48 ± 4.83	23.36 ± 8.99
***t*-test [t, P]**	t = 3.87, P = 0.022*	
**Practice**		
Pre-intervention	14.80 ± 2.96	14.36 ± 1.56
Post-intervention	15.49 ± 2.44	18.17 ± 2.54
***t*-test [t, P]**	t = 1.04, P = 0.357	

**t:***t*-test ***P*:** Significance * Significant (p ≤ 0.05).

Table 3 shows a relationship between of the mean and standard deviation of the attitude and practice of newly graduated nurses toward nursing care of preterm infants in the instructional guidelines and group discussion. According to newly graduated nurses' attitudes during pre-intervention and post-intervention, statistically significant differences were found for tube feeding, warmth, hygienic care, phototherapy care, and oxygen therapy (P = 0.046, P = 0.027, P = 0.036, P = 0.018, and P = 0.012, respectively). For the practice of new graduated nurses during pre-intervention and post-intervention, statistically significant differences were found between the instructional guidelines and group discussion only for hygienic care (P = 0.041), while no statistically significant differences were found in the practice of new graduated nurses toward nursing care of preterm infants in tube feeding, warmth, phototherapy care, and oxygen therapy (P = 0.278, P = 0.123, P = 0.078, and P = 0.089, respectively).

**Table 3:** Relationship between the mean and standard deviation of the attitude and practice of new graduated nurses toward nursing care of preterm infants in the instructional guideline and group discussion.

Main areas of focus for Preterm Infant Nursing Care	Attitude		Practice	
	Instructional Guideline	Group Discussion	Instructional Guideline	Group Discussion
**Tube feeding**				
Pre-intervention	5.80 ± 4.03	5.02 ± 2.46	4.22 ± 1.13	3.90 ± 0.92
Post-intervention	7.34 ± 3.72	10.01 ± 1.3	4.40 ± 1.01	5.22 ± 1.06
***t*-test [t, P]**	t = 2.19, P = 0.046*		t = 1.19, P = 0.278	
**Warmth**				
Pre-intervention	1.46 ± 3.06	2.51 ± 2.51	2.85 ± 0.92	2.74 ± 0.65
Post-intervention	7.23 ± 3.72	10.01 ± 1.42	2.95 ± 0.78	3.17 ± 0.84
***t*-test [t, P]**	t = 3.17, P = 0.027*		t = 1.35, P = 0.123	
**Hygienic care**				
Pre-intervention	4.01 ± 3.05	3.20 ± 2.37	1.38 ± 0.51	1.31 ± 0.48
Post-intervention	5.83 ± 2.41	7.26 ± 1.52	1.45 ± 0.51	1.62 ± 0.48
***t*-test [t, P]**	t = 1.01, P = 0.036*		t = 1.23, P = 0.041*	
**Phototherapy care**				
Pre-intervention	3.42 ± 3.41	1.92 ± 2.91	6.35 ± 1.53	6.39 ± 1.03
Post-intervention	5.71 ± 2.45	6.95 ± 1.55	6.66 ± 1.35	8.12 ± 1.23
***t*-test [t, P]**	t = 3.57, P = 0.018*		t = 0.78, P = 0.078	
**Oxygen therapy**				
Pre-intervention	1.47 ± 3.15	2.53 ± 2.50	2.84 ± 0.90	2.72 ± 0.67
Post-intervention	7.26 ± 3.73	10.00 ± 1.43	2.97 ± 0.75	3.15 ± 0.86
***t*-test [t, P]**	t = 4.27, P = 0.012*		t = 2.15, P = 0.089	

**t:***t*-test ***P*:** Significance * Significant (p ≤ 0.05).

## 5 Discussion

Both instructional guidelines and group discussion techniques significantly enhanced new graduates' attitudes toward caring for preterm infants. However, the rise in attitude ratings after group discussion was twice that of groups taught according to instructional guidelines. Consequently, it can be said that the group discussion method proved to be more effective.

In contrast, our results indicated that group discussion could significantly improve new nurses' preterm infant care practices but had no significant impact on instructional guidelines. Most of the research on the care of preterm infants has been descriptive or conducted with nursing staff [19].

There are few studies on the impact of instructional guidelines on the attitudes and practices of new nurses caring for preterm infants. However, this method is also commonly used in other research questions, with inconsistent results [21,22].

Several studies have shown that instructional guidelines can effectively influence patient decision-making [23] and medical practice [21]. However, it was found that this method did not affect nurses' knowledge, and nurses did not agree with this technique [19]. When properly designed and used for a specific purpose, instructional guidelines influence people's knowledge adequately. However, this method may not be very effective in practice with nursing staff and people, as nurses are less proactive and real relationships do not exist. Furthermore, active and deep reading is not guaranteed when using reading material for self-study [19].

Also, this study showed that group discussion was more effective than instructional guidelines in influencing new nurses' attitudes. This finding is consistent with several previous studies [24,25]. Group discussions will likely influence nurses' attitudes and practices in caring for preterm infants. Nurses must actively listen to and respect other people's views, think analytically about other people's views, and enthusiastically express appropriate and logical opinions. Her communication skills also improve as she has to react to, accept, or reject other ideas.

Pre-intervention, all nurses participating in the study had a low mean and standard deviation of the attitude and practice of newly graduated nurses toward nursing care of preterm infants in the instructional guidelines and group discussion. Several studies have reported that preterm infant care is not fully respected by medical and nursing teams [26,27]. Nursing curricula may not adequately address caring for preterm infants. Therefore, urgent measures are desired to overcome this problem.

Although new nurses' views on all facets of preterm baby care changed as a result of both interventions in this study, the group discussion showed to be more successful. But while no statistically significant differences were found in the practice of new graduated nurses toward nursing care of preterm infants in tube feeding, warmth, phototherapy care, and oxygen therapy, the curriculum approach is less affected in all aspects of new nurse practice. Group discussions are effective in learning various topics, but this method is rarely used to improve new nurses' attitudes and practices regarding preterm infant care. A group discussion was held just before entering the practice area for the newly graduated nurses who participated in this study to reflect on their prior performances and clinical experiences. Such considerations influenced their attitudes and may have prompted them to reconsider their behavior in the clinical setting. Although this study considered both the attitudes and practices of new nurses, it can be assumed that post-intervention observations may influence the attitudes and responses of new nurses to interventions.

Training programs through instructional guidelines and group discussion play a major role in how well the new nurse moves through the competency regarding nursing care, so training must be circulated through the studied mentorship and educational techniques for new nurses by nursing educational institutions and health care institutions to be a source of continuous guidance and direction for them to increase their mastery of quality nursing care in the future, especially for nurses of premature infants. Also well-designed, effective instructional guidelines and group discussion programs for newly graduated nurses based on their competency assessment and knowledge test upon arrival in the clinical setting. Putting educational materials from the orientation program in a booklet to be a guide for newly graduated nurses and providing them with a designed evaluation manual based on standard procedures included in the orientation booklet.

## 6 Limitations of the study

The sample size was relatively small but comparable to previous studies. Despite all efforts to standardize the educational intervention, the study's outcome may have been impacted by external factors, such as participant disparities in ability and ability to spread study messages. Additionally, in terms of bias risk, blinding outcome assessment or performance bias is lacking, as it is in most randomized controlled trials (RCTs), difficult to calculate sample size, and difficult to control confounding factors, which may have decreased reliance on study findings.

## 7 Implications and conclusions

In conclusion, newly graduated nurses exhibit low attitudes and practices regarding respect for premature nursing care at baseline. All interventions in this study enhanced newly graduated nurses' attitudes toward primary care. However, group discussion was more active in refining the practice of new nurses. Nursing educators are encouraged to use group discussion techniques to improve new nurses' attitudes and practices toward premature nursing care. Due to the small size of our sample, it is recommended that similar studies with larger samples be repeated. In addition, follow-up was short, and we had no say in how the syllabus was read by the students. Therefore, extended follow-up and some policies for carefully reading instructional guidelines are recommended.

In order to effectively transform mentoring efforts into skill development, this study emphasizes the need of imparting a compassionate experience through on-the-job mentorship to shape the information-seeking and information-sharing behaviors of new nurses. By matching mentorship duties to the job description and institutional performance management system, sustainability can be attained. Group discussion was shown to be a workable and successful method for nursing personnel in the current study, and, therefore, can serve as the main driving force when designing mentorship programs. Such an initiative requires new nurses to develop information-seeking skills and training that enhances their competencies.

### 7.1 Ethical approval and consent

This study adheres to the highest ethical standard, as the ethical committee approved the study protocol of the IRB at the University of Bisha with code number (UBCOM-RELOC H-06-BH-087)/0608.23). This study followed the ethical standards in the 1964 Declaration of Helsinki and its later amendments. The study was accepted by the Ethics Committee of the College of Applied Medical Sciences in Al-Namas at the University of Bisha, KSA. The study was also approved by the Ethics Committee of Al-Namas General Hospital and the Pediatric Hospital of Assiut University, Egypt. All new graduate nurses were informed of the purpose of the study and completed a written informed consent form prior to the study's beginning. They were also informed that although they would be monitored covertly, the findings would remain confidential and would not impact their clinical training performance.

## Consent for publication

Not applicable.

## Funding

The University of Bisha through the general research project under grant number (UB-GRP-48-1444) funded the study. The funders had no role in study design, data collection and analysis, decision to publish, or preparation of the manuscript.

## Disclosure statement

The authors disclose no competing interests. The writing and content of this article are the sole responsibility of the authors.

## Data availability

Data will be made available on request.

## CRediT authorship contribution statement

**Abdulaziz Asiri:** Formal analysis, Data curation, Conceptualization. **Faransa A. Ahmed:** Resources, Project administration, Methodology, Investigation. **Abeer A. Almowafy:** Writing – review & editing, Writing – original draft, Validation, Supervision, Software, Project administration, Methodology, Data curation, Conceptualization. **Rasha A. Mohamed:** Software, Resources, Project administration, Methodology. **Wael G. Nouh:** Supervision, Software, Resources, Project administration, Methodology. **Aml S. Abdelrahem:** Supervision, Software, Methodology, Investigation, Formal analysis, Data curation. **Rehab H. Kafl:** Visualization, Validation, Supervision, Software, Data curation, Conceptualization. **Manal F. Mohamed:** Writing – original draft, Visualization, Validation, Supervision. **Shimaa M. Moursy:** Writing – review & editing, Writing – original draft, Visualization, Methodology, Funding acquisition, Formal analysis.

## Declaration of competing interest

The authors declare that they have no known competing financial interests or personal relationships that could have appeared to influence the work reported in this paper.
