# Supplementary material for: Instructional guidelines and group discussion effects on new nurses’ competency regarding nursing care of preterm infants
Source: Heliyon. 2024 Jun 6;10(11):e32586. doi: 10.1016/j.heliyon.2024.e32586 (PMC11219499; doi:10.1016/j.heliyon.2024.e32586)
Supplement: Multimedia component 1 [file mmc1.pdf]

<u>Appendix (1)</u> Demographic Characteristics

- Nurse's Name (Not Obligatory): ……………… Age: ………………
- Gender: ……………. Marital Status: …………..
- Interesting In The Preterm Care Profession: Yes/No
- Conflict Due To Incompetence In Preterm Care In The Clinical Setting Of The NICU: Yes/No

<u>Appendix (2)</u> Newly Graduated Nurses' Attitudes about Nursing Care Competence for Preterm Infants domains

Nurses' Attitudes competence domains	Agree	No Idea	Disagree
1. **Attitudes About tube feeding competence domain.**			
− Discomfort from gavage tube placement can be minimized with competence practice.			
− It is the responsibility of nurses to be an advocate for competence in tube feeding for premature.			
− Competence in tube feeding is effective in reducing risks associated with vomiting.			
− I feel confident in my skills to safely facilitate gavage tube placement.			
− Competence in gavage tube placement can decrease oropharyngeal injures			
− I believe competence in gavage tube insertion facilities to oral feeding.			
2. **Attitudes About warmth competence domain.**	**Agree**	**No Idea**	**Disagree**
− I believe competence in premature warmth can eliminated heat loss within the first few minutes after birth.			
− Kept premature warm in the first 10-20 minutes.			
− Competence in premature warmth is effective in reducing risks associated with hypothermia.			
− I feel confident in my skills to safely facilitate premature warm.			
− It is promotes bonding and regulates a baby's temperature can promote heart rate and breathing.			
− I feel premature loses heat through its skin and respiratory tract to and I can promote the environment temperature.			
− It is the responsibility of nurses to be an advocate for competence in premature warmth.			
3. **Attitudes about hygienic care competence domain.**	**Agree**	**No Idea**	**Disagree**
− I think competence in premature hygiene care is important for premature health and comfort.			
− I feel premature bathing can reduce infection incidence.			
− I feel competence in premature changing diapers, is important for premature comfort.			
− I look for applying competence cleaning the ears and nose, clean only the surface of the ears and nose can prevent skin broken.			
− If clean the area carefully and gently and dry diaper rash through competence manner can rapidly healing.			
− I believe premature babies have weak resistance, and competence in body hygiene plays an important role.			
− I think my hands must be clean at all times, especially before holding premature babies.			
− I believe competence antiseptic technique is suboptimal in this high risk setting.			
4. **Attitudes about phototherapy care competence domain.**	**Agree**	**No Idea**	**Disagree**
− I think maximize skin exposure, so must dress the baby in a nappy and their protective eye covers only.			
− I think should daily fluid requirements should be reviewed and individualized for gestational and postnatal age.			
− I feel must maintain a strict fluid balance chart.			
− I believe breast feeds may need to be limited to 20 minutes if bilirubin level is high to minimize amount of time out of the lights			
− I believe must monitor vital signs and temperature at least 4 hourly.			
− I feel should cover lipid lines with light resistant, reflective tape to avoid peroxidation.			
5. **Attitudes about competence care during oxygen therapy domain.**	**Agree**	**No Idea**	**Disagree**
− I believe competence in premature care during oxygen therapy can save the child's life.			
− I believe invasive procedures & handling may increase the child's oxygen			

	consumption & lead to worsening hypoxaemia.			
–	I think oxygen therapy should be closely monitored & assessed at regular intervals			
–	I believe mustn’t smoke in the vicinity of oxygen equipment.			
–	I believe must turn off oxygen immediately when not in use because pool in fabric making the material more flammable.			
–	I think should never leave the nasal prongs or mask under or on bed coverings or cushions whilst the oxygen is being supplied.			
–	I feel oxygen cylinders should be secured safely to avoid injury.			
–	I believe mustn’t store oxygen cylinders in hot places.			

Appendix (3) Newly Graduated Nurses' Practice about Nursing Care Competence for Preterm Infants domains

Nurses' Practice competence domains	Compliance	Non-compliance
**1. Nurses' Practice competence about tube feeding domain.**		
− Collect the equipment you will need and wash your hands.		
− Check the feed to ensure that it is your EBM (expressed breast milk) and counter sign the feed chart and check the milk's temperature.		
− Check the nasogastric tube is inserted to the correct depth, open the tube cap and aspirate (suck out) any milk from the stomach with the empty syringe, the milk you have sucked out from the stomach is called the gastric residual, return the gastric residual (aspirated milk) via the feeding tube after checking with the nurse that you should do so.		
− Administer feed over the specific time interval, close the feed cap or, if the baby is on a ventilator or CPAP, leave the tube uncapped, attach an open syringe and suspend it to provide a release for air, leave baby in a settled position.		
− Dispose of used equipment and wash your hands with reporting and recording.		
**2. Nurses' Practice competence about warmth domain.**		
− Apply skin-to-skin care and effectively be kept warm by placing them naked against the mother's bare breasts. The infant should wear a woolen cap and nappy.		
− Put the premature infant in a closed incubator, a temperature can be carefully controlled. Warm the nursery and the infant should be dressed to prevent heat loss by radiation.		
− Place premature under the radiant warmers (overhead radiant heaters), when resuscitating an infant or for nursing a very sick infant in an intensive care unit.		
− Warm and dry the premature infant before dressing.		
**3. Nurses' Practice competence about hygienic care domain.**		
− Keeping baby's genitals clean helps to prevent infections and use warm water to clean baby genitals, mild cleanser and a cotton ball or soft cloth.		
− Clean baby eyes, baby ears and baby noses with warm water and a cloth or cotton wool when you give your baby a bath.		
− Applying cleaning the ears and nose, clean only the surface of the ears and nose to prevent skin broken.		
− Clean and gently and dry diaper rash through competence manner		
− Clean hands at all times, especially before holding premature babies.		
− Promote clean cord care and reducing harmful cord applications, dry cord care and use of soap and water solution to clean the cord if visibly soiled		
**4. Nurses' Practice competence about phototherapy domain**.		
− Dress the baby in a nappy and their protective eye covers only.		
− Review daily fluid requirements and individualized for gestational and postnatal age.		
− Maintain a strict fluid balance chart.		
− Monitor vital signs and temperature at least 4 hourly.		
**5. Nurses' Practice competence about oxygen therapy domain**.		
− Closely monitored & assessed oxygen at regular intervals		
− Turn off oxygen immediately when not in use because pool in fabric making the material more flammable.		
− Never leave the nasal prongs or mask under or on bed coverings or cushions whilst the oxygen is being supplied.		
− Oxygen cylinders should be secured safely to avoid injury.		
